# Mediation pathways and effects of green structures on respiratory mortality via reducing air pollution

**DOI:** 10.1038/srep42854

**Published:** 2017-02-23

**Authors:** Yu-Sheng Shen, Shih-Chun Candice Lung

**Affiliations:** 1 Research Center for Environmental Changes, Academia Sinica, Taipei, Taiwan; 2 Department of Atmospheric Sciences, National Taiwan University, Taipei, Taiwan; 3 Institute of Environmental Health, National Taiwan University, Taipei, Taiwan

## Abstract

Previous studies have shown both health and environmental benefits of green spaces, especially in moderating temperature and reducing air pollution. However, the characteristics of green structures have been overlooked in previous investigations. In addition, the mediation effects of green structures on respiratory mortality have not been assessed. This study explores the potential mediation pathways and effects of green structure characteristics on respiratory mortality through temperature, primary and secondary air pollutants separately using partial least squares model with data from Taiwan. The measurable characteristics of green structure include the largest patch percentage, landscape proportion, aggregation, patch distance, and fragmentation. The results showed that mortality of pneumonia and chronic lower respiratory diseases could be reduced by minimizing fragmentation and increasing the largest patch percentage of green structure, and the mediation effects are mostly through reducing air pollutants rather than temperature. Moreover, a high proportion of but fragmented green spaces would increase secondary air pollutants and enhance health risks; demonstrating the deficiency of traditional greening policy with primary focus on coverage ratio. This is the first research focusing on mediation effects of green structure characteristics on respiratory mortality, revealing that appropriate green structure planning can be a useful complementary strategy in environmental health management.

Air pollution and high temperature are significant public health threats, particularly in urban areas with intensive human activities under the trend of climate change. Many studies showed that increased morbidity and mortality of respiratory diseases are associated with high temperature^1^,^2^,^3^,^4^,^5^,^6^ and various air pollutants, especially ozone, particulate matter, nitrogen oxide, and sulfur dioxide^7^,^8^,^9^,^10^,^11^. Indeed, the risks of respiratory diseases are not only affected by environmental factors, but also by hereditary and lifestyle. However, environmental factors can be improved by public policy which offers opportunity to reduce risks of respiratory mortality. This study focuses on green spaces, an important environmental aspect, which could be designed by appropriate urban planning.

Green spaces serve environmental functions of moderating temperature^12^,^13^,^14^,^15^,^16^ and reducing air pollutants^15^,^17^,^18^,^19^,^20^. For instance, Bowler *et al*.^12^ indicated that urban park was cooler than a non-green site (approximately 1 °C); Jim and Chen^17^ showed that urban trees at Guangzhou removed 312.03 mg of air pollutants in 2000. Owing to practical problems involved in reducing air pollution and temperature increase from their sources, appropriate management of green spaces can serve as a complementary strategy for lowering risks of respiratory diseases caused by air pollution and temperature increase.

Previous research on green spaces and health focused mainly on enhancing residents’ psychological health (such as improving the happiness or reducing mental illness)^21^,^22^,^23^,^24^,^25^,^26^ and physical health by promoting physical activities^12^,^27^,^28^. Some of these studies explored the association between respiratory diseases and green spaces^29^,^30^,^31^,^32^,^33^,^34^, but their findings were inconsistent and the mediation effects of green spaces on mortality of respiratory diseases had not been assessed. Additionally, the spatial scale of analysis in previous studies was mostly limited to the community scale; and green spaces in large metropolitan had rarely been analyzed. For green space to serve its environmental function, it should cover extensive land areas, thus such analyses should include metropolitan areas to produce more reliable and valid results. Prior researches emphasized total green area or green space coverage ratio only, and ignored the characteristics of green structures which may also play a role in effective green space planning for ameliorating risks of respiratory mortality. The value of green structures on mediating cardiovascular mortality has been demonstrated by Shen and Lung^35^. This study investigated the mediation effects of green structures on respiratory mortality and explored critical green structure factors.

Green structures are defined as spatial structures and contexts of green spaces by urban planners. This investigation focused on the measurable characteristics of green structures within metropolitan areas, including the largest patch percentage, landscape proportions, aggregation, patch distance, and fragmentation. The objective was to assess the potential mediation effects of green structures on mortality of respiratory diseases through reducing air pollution (levels of primary and secondary air pollutants) and lowering temperature, and to further identify important green structure factors for references by urban planners in achieving effective green space planning to improve public health. Data from Taiwan were employed to construct an empirical model. The methodology could also be applied to other countries with available data to evaluate green structure characteristics in order to obtain practical strategies to reduce health risks in their own countries.

## Results

### Model evaluation

For the partial least squares (PLS) model, the goodness-of-fit (GoF) index calculated from the geometric mean of the average communality index and the average R^2^ value^36^ was 0.585. Wetzels *et al*.^37^ indicated that the proper GoF index of PLS models with large sample size (the sample size of our model was 10,000) was 0.36. Thus, our PLS model had proper GoF. To verify the validity and reliability of the outer model, the composite reliability (CR) and average variance extracted (AVE) of latent variables exceeded 0.7 and 0.5, respectively (Table 1), indicating that the PLS model had convergent validity, with highly related observed variables measuring the same latent variables^38^,^39^. The square roots of the AVE for each latent variable were all greater than the correlation coefficients among the latent variables, indicating that this model had discriminating validity for differentiating the latent variables^40^. Additionally, the CR and Cronbach’s α of each latent variable exceeded the criteria (0.7) of construct reliability^38^,^41^ (Table 1), showing that the latent variables had internal consistency.

### Path diagrams

**Figure 1:**
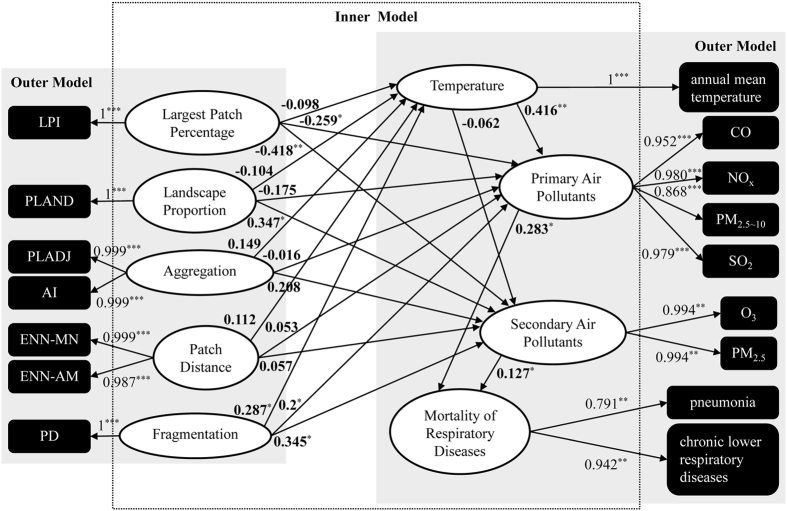
PLS path diagrams of green structures and atmospheric environment on mortality of respiratory diseases. Note: *p < 0.05, **p < 0.01, ***p < 0.001.

Figure 1 shows PLS path diagrams of green structures and atmospheric environment (temperature, primary air pollutants, and secondary air pollutants) for mortality of respiratory diseases. In the outer model, the loadings and paths revealed the quantitative relationships between the latent and observed variables. All loadings of the observed variables exceeded 0.7 and all findings were statistically significant (*p* < 0.05), indicating that the observed variables reflected accurately the meanings of the latent variables. For instance, measurement of primary air pollutants in terms of CO, NO_x_, SO_2_ and PM_2.5–10_ concentrations had loadings of 0.952, 0.98, 0.979, and 0.868, respectively, indicating that CO, NO_x_, SO_2_ and PM_2.5–10_ were accurate indicators of primary air pollutants with NO_x_ being the most representative.

For the inner model, the path explained the relationships between the latent variables, and the standardized path coefficient quantified the effect of the corresponding path. The effect of the largest patch percentage on mortality of respiratory diseases included the path through primary air pollutants (largest patch percentage ^(−0.259)^> primary air pollutants ^(0.283)^> mortality of respiratory diseases), and secondary air pollutants (largest patch percentage ^(−0.418)^> secondary air pollutants ^(0.127)^> mortality of respiratory diseases). The landscape proportion also affected mortality of respiratory diseases through secondary air pollutants (landscape proportion ^(0.347)^> secondary air pollutants ^(0.127)^> mortality of respiratory diseases). Moreover, the mediation effect of fragmentation on mortality of respiratory diseases had three pathways, namely through temperature and primary air pollutants successively (fragmentation ^(0.287)^> temperature ^(0.416)^> primary air pollutants ^(0.283)^> mortality of respiratory diseases), through primary air pollutants (fragmentation ^(0.2)^> primary air pollutants ^(0.283)^> mortality of respiratory diseases), and through secondary air pollutants (fragmentation ^(0.345)^> secondary air pollutants ^(0.127)^> mortality of respiratory diseases). Thus, fragmentation has mediation effects on respiratory mortality via all three atmospheric environment attributes. In addition, the largest patch percentage mediates respiratory mortality via primary and secondary air pollutants while landscape proportion mediates respiratory mortality via only secondary air pollutants.

Path coefficients also indicate whether the mediation effect is positive or negative. Primary and secondary air pollutants were both positively and directly associated with mortality of respiratory diseases; on the other hand, temperature did not have direct effect on mortality of respiratory diseases; it only had significant indirect positive effect through primary air pollutants. In the relationship between green structure and mortality of respiratory diseases, largest patch percentage had significant negative effect on both primary and secondary air pollutants, and then indirect positive relationship with mortality of respiratory diseases through primary and secondary air pollutants. Landscape proportion was only positively associated with secondary air pollutants, and had indirect positive influence on mortality of respiratory diseases via secondary air pollutants. Fragmentation significantly and positively affected temperature, primary air pollutants, and secondary air pollutants, and had positive impact on mortality of respiratory diseases. While the path diagrams detail the influences and effects of individual variables, they do not show the total effects. Thus, the total effects of green structures and atmospheric environment on mortality of respiratory diseases are given in Table 2 and described in the following subsection.

### Total effects

Total effect calculated by standardized path coefficient is unitless. Table 2 shows that largest patch percentage, fragmentation, primary air pollutants, and secondary air pollutants all had significant impact on mortality of respiratory diseases. Largest patch percentage reduced the mortality of pneumonia and chronic lower respiratory diseases by altering the levels of primary and secondary air pollutants; the total decreased effect was −0.131. In contrast, fragmentation had enhanced mediation effects on mortality of pneumonia and chronic lower respiratory diseases through temperature, primary air pollutants, and secondary air pollutants; the total increased effects were 0.112, indicating that fragmentation of green space contributed to raise respiratory mortality. Moreover, the total increased effects of primary and secondary air pollutants on mortality of pneumonia and chronic lower respiratory diseases were 0.283 and 0.127, respectively.

In view of the different characteristics of primary and secondary air pollutants, this study purposefully analyzed the mediation effects of green structure on primary and secondary air pollutants separately. As revealed by the total effects of green structures on primary air pollutants (Table 3), fragmented green spaces increase while the largest patch percentage reduces levels of primary air pollutants. Similarly, a high proportion of fragmented green spaces increase while the largest patch percentage reduces levels of secondary air pollutants. Additionally, the reduction effect of the largest patch percentage on secondary air pollutants (−0.359) is slightly greater than that on primary air pollutants (−0.3).

## Discussion

Green structures contribute to enhance environmental beauty^42^ and stabilize ecological systems^43^. The empirical results obtained that green structures also have several public health benefits. First, the largest patch percentage of green structures reduced mortality of pneumonia and chronic lower respiratory diseases through decreasing primary and secondary air pollutants, while fragmentation of green structures enhanced mortality of pneumonia and chronic lower respiratory diseases by increasing temperature, primary and secondary air pollutants. Compared with previous studies which assessed only the effects of total area or coverage ratio of green space on incidence of respiratory diseases through mediating physical activities^27^,^44^,^45^,^46^, this investigation is the first to analyze the effect of “green structures” on respiratory mortality, to consider the mediation effects of green structures through various atmospheric environment attributes, and to report on the complex relationships between green structures, atmospheric environment, and respiratory mortality. The present results not only demonstrate the mediation pathways and beneficial effects of minimizing fragmentation and maximizing the largest patch percentage of green structures, but also highlight the crucial importance of these green structure indicators in greening policy for enhancing public health.

Secondly, according to the total effect of each green structure variable on air pollution, the two critical green structure indicators influencing primary and secondary air pollutants are largest patch percentage and fragmentation. Additionally, both fragmentation and landscape proportion would increase secondary air pollutants. In other words, too many fragmented and small-sized green patches of land are actually enhancing the levels of secondary air pollutants. Previous studies had shown that trees emit volatile organic compounds (VOCs) which are precursors of secondary air pollutants including O_3_ and PM_2.5_^47^,^48^,^49^; meanwhile, green spaces can also block secondary air pollutants^17^,^18^,^19^. It is speculated that the high proportion of fragmented and small-sized green spaces have large precursor-emission areas but are weak in blocking air pollutants because the blocking effect may be more obvious in a large green space. Hence, both landscape proportion and fragmentation had positive association with secondary air pollutants. Moreover, this finding also demonstrates the deficiency of traditional greening policy that emphasizes only total areas and coverage ratio; increase in total area and number of small-sized green patches of land is actually aggravating rather than ameliorating air pollution. The results revealed that reducing fragmentation and increasing the largest patch percentage of green structures should be mainstreamed into the greening policy to reduce air pollution for public health benefit. Green structure characteristics should replace total areas and coverage ratio as the focuses of green planning.

Thirdly, although respiratory mortality has shown to be associated with temperature and air pollution both individually and in combination^1^,^2^,^6^,^7^,^9^,^10^,^11^, this study considered temperature and air pollution (primary and secondary air pollutants) simultaneously. In addition to confirming that primary and secondary air pollutants indeed enhanced respiratory mortality, the analysis on relative importance of their mediation pathways (Fig. 1) found that primary air pollutants have greater influence on mortality of respiratory diseases than secondary air pollutants.

Additionally, The relationships of air pollution, respiratory mortality, and green area might be different in different season because of different weather condition (such as rainfall, solar radiation, humidity and so on). The effects of different seasons could not be evaluated because the data of all variables in this study were annual mean. Those seasonal effects could be evaluated in future studies.

In terms of whether other variables should be considered in our PLS model, we have explored other alternatives such as confounding variables which are the extraneous variable that correlates with both the dependent variable and the independent variable. There was no confounding variable which correlated with both green structures (independent variables) and respiratory mortality (dependent variable). Thus, our PLS model did not consider confounding variables.

Finally, this study evidences the benefits of green structures on public health, especially in reducing the mortality of respiratory diseases. The findings would serve as useful references for greening policy formulation and for public health promotion. The public should be educated or made aware of the health benefits of maximizing the largest green patch percentage and minimizing fragmentation of green spaces, which may motivate them to achieve that in their communities and living environment. Furthermore, this study also demonstrates that PLS modeling is effective in assessing the total effects and relative importance of various pathways of influential factors on health outcomes. Thus, PLS modeling can be further applied in other health studies to analyze complex relationships of health outcome with their determinants.

This study provides a new perspective that proper management of structure determinants of green space lowers the mortality of pneumonia and chronic lower respiratory diseases. This study also shows that maximizing the largest green patch percentage and minimizing green space fragmentation are the most important factors for achieving health benefits. Additionally, too many small-sized green spaces would increase the levels of secondary air pollutants; thus, the greening policy for health benefits should focus on green structure characteristics, instead of total area of green spaces.

## Methods and Data

### Methodology

PLS modeling was used in this study to clarify the complex effects and relationships of green structures, atmospheric environment (i.e., temperature, primary air pollutants, and secondary air pollutants), and mortality of respiratory diseases. PLS modeling has three major advantages. The first is that it can analyze the relationships between multiple dependent and independent variables and compare mediation effects simultaneously. Secondly, it minimizes the biases of interference, multicollinearity, missing data, and the assumption of sample distribution. The third advantage is that a PLS model has stronger predictive power than a regression model^50^.

PLS modeling is a type of structural equation modeling that involves using an outer model and inner model to represent a causal network of latent variables^40^,^50^. The outer model describes the relationships between latent variables and observed variables, while the inner model describes the relationships between endogenous and exogenous latent variables. In this study, the endogenous latent variable evaluated is influenced by the exogenous latent variables in the inner model. The algorithm includes two steps, the first step is iterative estimation of latent construct scores, and the second step is the final estimation of coefficients of the PLS model. The detailed methodology and algorithm can be found in previous publications^40^,^50^. The inputs of dependent and independent variables are standardized. Thus, the obtained path coefficient and total effect estimates are standardized coefficients allowing comparison of impacts among different independent variables. The unitless standardized path coefficient denotes the extent of standard deviation change of endogenous latent variable attributed to one standard deviation change of exogenous latent variable. The unitless total effect estimates are the sum of all standardized path coefficients with similar statistical meanings. In this work, SmartPLS (SmartPLS GmbH, Bönningstedt, Germany), widely employed in engineering, social sciences and psychology, is used for PLS model construction and data analysis.

### Indicators

Table 4 lists the definitions of latent variables and observed variables which are classified into three categories. First, the green structure category comprises five latent variables: largest patch percentage, landscape proportion, aggregation, patch distance, and fragmentation^51^,^52^. The observed variables are indicators from landscape metrics^51^,^52^, which were adopted to evaluate green structures. The detailed methodology and formulas employed for these indicators are listed in Supplementary Information Table S1. In brief, largest patch index (LPI) (%) denotes the percentage of the largest patch area in a specific area. LPI is adopted for analyzing the scale of the largest green patch in this study. Percentage of landscape (PLAND) (%) of green structure is the percentage of landscape composed of the green patch type. This study adopts PLAND to analyze the percentage of green space in a specific area. Percentage of like adjacencies (PLADJ) (%) is calculated using the percentage of patch adjacencies involving the corresponding patch type with similar adjacencies, and aggregation index (AI) (%) is calculated by an adjacency matrix, which shows the frequency for different pairs of patch types to appear side-by-side on the map. Both PLADJ and AI are used for measuring the patch aggregation in the green space. Area-weighted mean nearest-neighbor distance (ENN-AM) (meters) indicates the area-weighted patch distance between patches, and mean nearest neighbor distance (ENN-MN) (meters) refers to the distance between each patch. Both ENN-AM and ENN-MN are adopted for measuring the dispersion of green spaces. Patch density (PD) (number per 100 hectares) is the ratio of the number of patches in a specific area, and PD is used to analyze the fragmentation of green spaces in this study^51^,^52^. In summary, the green structure characteristics of scale, coverage ratio, aggregation, dispersion, and fragmentation are analyzed by the aforementioned variables.

In this study, the atmospheric environment category comprises three latent variables: temperature, primary air pollutants, and secondary air pollutants. The observed variable for the latent variable of temperature is the mean annual temperature. Primary air pollutants refers to the levels of various primary air pollutants, such as carbon monoxide (CO), nitrogen oxide (NO_x_), sulfur dioxide (SO_2_) and particulate matter with particle size ranging from 2.5 to 10 microns (PM_2.5–10_)^53^,^54^,^55^,^56^. Secondary air pollutants refers to the levels of various secondary air pollutants, such as ozone (O_3_) and particulate matter with particle size below 2.5 microns (PM_2.5_)^53^,^54^,^55^,^56^. It should be emphasized that primary pollutants are pollutants emitted directly from a source; while secondary pollutants are formed as a result of chemical reactions of pollutants in the atmosphere. Nevertheless, PM_2.5_ could be formed in both primary and secondary processes, the main composition of primary aerosols were elemental carbon and primary organic carbon, and the main composition of secondary aerosols were sulfate, nitrate, and secondary organic carbon. The ratio of secondary aerosols to primary aerosols in the Taipei Metropolitan Area based on previous studies^57^,^58^ was 1.9; the detailed calculation was in Supplementary Information. Thus, PM_2.5_ is classified as secondary air pollutants in the current model because it comprises a significant portion of secondary components. Primary and secondary air pollutants might have different impacts on mortality of respiratory diseases and be affected by green structures in different ways; hence, air pollutants in this study are divided into these two subcategories, which is a distinct feature compared with previous studies.

Finally, the latent variable of the health outcome category was respiratory mortality. According to the international statistical classification of diseases and related health problems 10^th^ revision (ICD-10) from the Ministry of Health and Welfare, respiratory mortality (standardized by age distribution at 2000 from World Health Organization) includes death due to acute bronchitis and bronchiolitis, pneumonia, and chronic lower respiratory diseases. However, the district database contained too many zero values regarding the mortality of acute bronchitis and bronchiolitis. Hence, the observed variables for respiratory mortality included only the mortality rate of pneumonia and chronic lower respiratory diseases.

### Data

**Figure 2:**
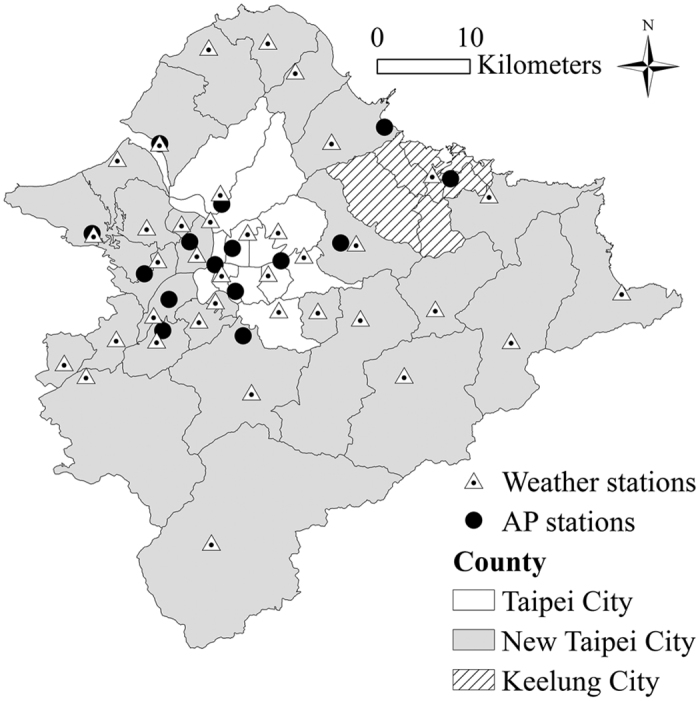
Distribution of weather and air pollution stations in Taipei Metropolitan Area. Locations of the weather and AP stations were from Central Weather Bureau and Environmental Protection Administration (open data), respectively. Boundary of districts was from National Land Surveying and Mapping Center (open data). This original map was created by ArcGIS 10.3 software (http://www.esri.com/software/arcgis/).

This study took the 48 administrative districts of the Taipei Metropolitan Area as an empirical case. The Taipei Metropolitan Area comprises Taipei City, New Taipei City, and Keelung City (Fig. 2), and covers an area of approximately 2,457km^2^. Moreover, the area of districts ranges from 4.3 to 321.2 km^2^ with an average of 51.2 ± 59.9 km^2^. Geographically, the Taipei Metropolitan Area is located in a basin, which easily accumulates air pollution and heat. Mountainous areas are present on the periphery of the Taipei Metropolitan Area and small neighborhood parks are scattered in the core urban areas. This feature results in different green structure characteristics among different districts. Additionally, the Taipei Metropolitan Area has a high urbanization level and high population densities in its core urban areas. The population density of districts ranges from 16.6 to 41250.5 person/km^2^ with an average of 8,485 ± 10,089 person/km^2^.

Related numerical data collected were those of year 2010. Data for the observed variables of the green structure category in 48 districts were collected from the National Land-Use Survey of National Land Surveying and Mapping Center, and indicators of landscape metrics were calculated using the software of FRAGSTATS 3.3, which was developed by Dr. Kevin McGarigal of University of Massachusetts (Massachusetts, USA) and widely employed by researchers in landscape ecology, urban planning, and forestry. Temperature data of districts were obtained from 38 Central Weather Bureau sites which covered most of the Taipei Metropolitan Area (Fig. 2). Data of primary and secondary air pollutants of districts were obtained from the 15 Environmental Protection Administration stations located in densely populated districts (Fig. 2). Because the air pollutants are almost uniformly distributed in the Taipei Metropolitan Area which is in a basin^59^, the bias between real value and interpolation should be minimal; the same applies in temperature. Thus, data for those districts with missing values for primary air pollutants, secondary air pollutants, and temperature were interpolated through the ordinary kriging method from values measured by stations located in other districts. The data of respiratory mortality, according to adjudicated death certificates, were aggregated by districts and collected from the database of the Ministry of Health and Welfare. Table 5 shows the descriptive statistics of the data collected. PLS model was employed to analyze the relationships of respiratory mortality in districts with the corresponding green structure, air pollution, and temperature. In view of the small sample size, this work adopted the bootstrap resampling method^60^ to generate 10,000 samples for estimating all parameters and for constructing the PLS model.

## Additional Information

**How to cite this article**: Shen, Y.-S. and Lung, S.-C.C. Mediation pathways and effects of green structures on respiratory mortality via reducing air pollution. *Sci. Rep.* **7**, 42854; doi: 10.1038/srep42854 (2017).

**Publisher's note:** Springer Nature remains neutral with regard to jurisdictional claims in published maps and institutional affiliations.

## Supplementary Material

Supplementary Information

## Figures and Tables

**Table 1:** Results of PLS model evaluation.

Characteristic	Latent Variables	AVE^a^	CR^b^	Cronbach’s α
Green Structures	Largest Patch Percentage	1.000	1.000	1.000
	Landscape Proportion	1.000	1.000	1.000
	Aggregation	0.999	0.999	0.999
	Patch Distance	0.986	0.993	0.989
	Fragmentation	1.000	1.000	1.000
Atmospheric Environment	Temperature	1.000	1.000	1.000
	Primary Air Pollutants	0.895	0.971	0.960
	Secondary Air Pollutants	0.988	0.994	0.988
Health Outcome	Mortality of Respiratory Diseases	0.803	0.909	0.899

Note: ^a^AVE means average variance extracted. ^b^CR means composite reliability.

**Table 2:** Total effects of green structures and atmospheric environment on mortality of respiratory diseases.

Exogenous latent variable	Endogenous latent variable	Total effect
Green Structures		
Largest Patch Percentage	Mortality of respiratory diseases	−0.131*
Landscape Proportion	Mortality of respiratory diseases	−0.010
Aggregation	Mortality of respiratory diseases	0.028
Patch Distance	Mortality of respiratory diseases	0.027
Fragmentation	Mortality of respiratory diseases	0.112*
Atmospheric Environment		
Temperature	Mortality of respiratory diseases	0.041
Primary Air Pollutants	Mortality of respiratory diseases	0.283*
Secondary Air Pollutants	Mortality of respiratory diseases	0.127*

**Table 3:** Total effects of green structures on primary and secondary air pollutants.

Exogenous latent variable	Endogenous latent variable	Total effect
Green Structures		
Largest Patch Percentage	Primary air pollutants	−0.300**
Landscape Proportion	Primary air pollutants	−0.218
Aggregation	Primary air pollutants	0.046
Patch Distance	Primary air pollutants	0.099
Fragmentation	Primary air pollutants	0.319**
Green Structures		
Largest Patch Percentage	Secondary air pollutants	−0.359**
Landscape Proportion	Secondary air pollutants	0.410**
Aggregation	Secondary air pollutants	0.118
Patch Distance	Secondary air pollutants	−0.010
Fragmentation	Secondary air pollutants	0.172*

Note: *p < 0.05, **p < 0.01, ***p < 0.001.

**Table 4:** Latent and observed variables of PLS model.

Characteristic	Latent Variables	Observed Variables
Green Structures	Largest Patch Percentage	⋅ Largest Patch Index (LPI)
	Landscape Proportion	⋅ Percentage of Landscape (PLAND)
	Aggregation	⋅ Percentage of Like Adjacencies (PLADJ)⋅ Aggregation Index (AI)
	Patch Distance	⋅ Mean Nearest Neighbor Distance (ENN-MN) ⋅ Area-Weighted Mean Nearest Neighbor Distance (ENN-AM)
	Fragmentation	⋅ Patch Density (PD)
Atmospheric Environment	Temperature	⋅ Mean annual temperature
	Primary Air Pollutants	⋅ CO levels
		⋅ NO_x_ levels
		⋅ SO_2_ levels
		⋅ PM_2.5-10_ levels
	Secondary Air Pollutants	⋅ O_3_ levels
		⋅ PM_2.5_ levels
Health Outcome	Mortality of Respiratory Diseases	⋅ Standardized mortality rate of pneumonia
		⋅ Standardized mortality rate of chronic lower respiratory diseases

**Table 5:** Descriptive statistics of observed variables (N = 48).

Observed Variables	Mean	Standard Deviation	Minimum	Maximum
LPI (%)	50.78	31.41	1.27	98.35
PLAND (%)	57.95	29.01	5.01	98.35
PLADJ (%)	82.48	12.89	36.70	98.52
AI (%)	83.91	12.01	41.07	98.79
ENN-MN (meters)	140.68	36.86	100.00	257.81
ENN-AM (meters)	108.11	17.13	100.00	182.84
PD (number per 100 hectares)	2.56	2.05	0.02	8.68
Mean annual temperature (°C)	22.94	1.64	17.10	24.70
CO (ppm)	0.596	0.23	0.24	1.26
NO_x_ (ppb)	29.01	14.74	5.20	71.78
PM_2.5-10_ (μg/m^3^)	21.18	7.07	0.87	39.00
SO_2_ (ppb)	19.79	7.83	3.90	34.42
O_3_ (ppb)	25.88	3.72	19.98	37.18
PM_2.5_ (μg/m^3^)	26.71	3.86	18.83	35.00
Mortality rate of pneumonia (per 100,000 people)	22.98	8.58	9.25	52.43
Mortality rate of chronic lower respiratory diseases (per 100,000 people)	11.42	5.05	1.00	27.95
