# Supplementary material for: Mediation pathways and effects of green structures on respiratory mortality via reducing air pollution
Source: Sci Rep. 2017 Feb 23;7:42854. doi: 10.1038/srep42854 (PMC5322332; doi:10.1038/srep42854)
Supplement: Supplementary Information [file srep42854-s1.pdf]

Mediation pathways and effects of green structures on respiratory mortality via reducing air pollution

Yu-Sheng Shen [a] Shih-Chun Candice Lung[a, b, c, *]

[a] Research Center for Environmental Changes, Academia Sinica, Taipei, Taiwan.
[b] Department of Atmospheric Sciences, National Taiwan University, Taipei, Taiwan
[c] Institute of Environmental Health, National Taiwan University, Taipei, Taiwan

[*]Corresponding author, Research Center for Environmental Changes, Academia Sinica, No. 128, Sec. 2, Academia Rd, Nangang, Taipei, Taiwan 11529; Tel: 886-2-26539885 ext. 277, Fax: 886-2-27833584, Email: sclung@rcec.sinica.edu.tw.

Supplementary Information

1.Indicators of landscape metrics

Table S1. Indicators of landscape metrics (class level)

Indicator	Formula	Unit
Percentage of Landscape (PLAND)	$\left(\sum_{j=1}^{n} a_{ij} \Big/ A\right)(100)$<br>$a_{ij}$: area of patch *j* (class *i*).<br>*A*: total landscape area.	%
Aggregation Index (AI)	$\left[g_{ss}/\left(\max \to g_{ss}\right)\right](100)$<br>$g_{ss}$: number of like adjacencies between pixels of patch type (class) *s* obtained using the single-count method.<br>max→g_{ss}: maximum number of like adjacencies between pixels of patch type (class) *s* obtained using the single-count method.	%
Percentage of Like Adjacencies (PLADJ)	$\left(g_{ii} \Big/ \sum_{k=1}^{m} g_{ik}\right)(100)$<br>$g_{ii}$: number of like adjacencies between pixels of patch type (class) *i* obtained using the double-count method.<br>g_{ik}: number of adjacencies between pixels of patch types (classes) *i* and *k* obtained using the double-count method.	%
Patch Density (PD)	$(n_i/A)(10{,}000)(100)$<br>n_i: number of patches in the landscape of patch type (class) *i*.<br>*A*: as previously defined.	Number per 100 hectares
Mean Nearest Neighbor Distance (ENN_MN)	$\sum_{j=1}^{n'} h_{ij} \Big/ n'_i$<br>$h_{ij}$：distance between patch *j* (class *i*) and patch of the corresponding class.<br>n'_i：number of patches in the landscape of patch type (class) *i* with nearest neighbor distance.	Meters
Area-Weighted Mean Nearest Neighbor Distance (ENN_AM)	$\sum_{j=1}^{n}\left[h_{ij}\left(a_{ij} \Big/ \sum_{j=1}^{n} a_{ij}\right)\right]$<br>$h_{ij}$, a_{ij}：as defined above.	Meters
Largest Patch Index (LPI)	$(MAX\ (a_{ij})/A)(100)$<br>a_{ij}, *A*: as defined above.	%

Source: Leitão et al. (2006); McGarigal and Marks (1995).

2.Calculation of the ratio of secondary aerosols to primary aerosols

The detail calculation of secondary aerosols/primary aerosols includes four steps. Those steps are as follow:

Step 1: Obtaining the SOC/OC and POC/OC from results of Chou et al. (2010).

SOC/OC = SOC/(SOC+POC) = five-years mean of SOC/(five-years mean of SOC+ five-years mean of POC) = 2.12/(2.12+4.78) ≒ 0.307

POC/OC = 1－(SOC/OC) ≒0.693

Step 2: Using the SOC/OC and POC/OC ratios to calculate the approximate concentration of SOC and POC in OC component in Chang et al. (2010)

SOC = 0.307×5 = 1.535

POC = 0.693×5 = 3.465

Step 3: Summing up the primary aerosols from EC and POC, and summing up the secondary sources from SOC, SO_4^{2-}, and NO_3^- in Chang et al. (2010)

primary aerosols = EC+POC = 1.6+3.465 = 5.065

secondary aerosols = SOC+SO_4^{2-}+NO_3^- = 1.535+6.4+1.8 = 9.735

Step 4: Calculating the ratio of secondary aerosols to primary aerosols

secondary aerosols/primary aerosols = 9.735/5.065 = 1.9
